# A Case of Koch's Spine Treated with Modified Transpedicular Vertebral Curettage and Posterior Fixation: A Novel Technique

**DOI:** 10.7759/cureus.915

**Published:** 2016-12-06

**Authors:** Paresh Golwala, Chirag Kapoor, Malkesh Shah, Aditya Merh, Ankur Kansagra

**Affiliations:** 1 Orthopaedics, Sumandeep Vidyapeeth, Vadodara, Gujarat

**Keywords:** spinal tuberculosis, transpedicular, decompression

## Abstract

Tuberculosis (TB) is a chronic granulomatous infection caused by acid-fast mycobacterium tuberculosis bacilli. Spinal involvement occurs in less than one percent of TB. Spinal TB (Pott’s disease) accounts for 50% of skeletal TB. Though it most commonly affects the thoracolumbar junction, it can occur at any level of the spine. Early diagnosis and treatment is mandatory in order to avoid neurological complications and spinal deformity. We report a case of a young female with tuberculosis of D12-L1 who was treated with posterior decompression using a modified transpedicular approach and posterior instrumentation with a successful outcome.

## Introduction

Tuberculosis (TB) is a chronic granulomatous infection, and spinal involvement is a destructive form of TB [1-2]. Spinal TB (Pott’s disease) accounts for 50% of skeletal TB and is more common in children and young adults [3]. It most commonly affects the thoracolumbar junction [4]. Spinal TB is also known to have neurological complications in about 10%–43% of the cases [5]. Early diagnosis and treatment is mandatory in order to avoid neurological complications and spinal deformity.

With the use of modern antitubercular drugs and rest and mobilization with suitable orthosis, biological control of the disease can be achieved along with a better quality of life as well as improved function of the joint involved. Surgery is indicated if the diagnosis is uncertain, an extensively spread lesion with or without abscess, a potentially unstable spine with or without neurological deficit which should be stabilized and for refractory disease in an adult and in children when kyphosis is likely to progress with growth [6].

We report a case of a young female with tuberculosis of the spine who was treated with a different technique of modified transpedicular vertebral curettage with a successful outcome.

## Case presentation

A 25-year-old female presented with an eight-month history of low back pain radiating to the left lower limb. The pain was associated with an evening rise of temperature and loss of appetite. She was not able to stand or walk for the past 15 days. She had a history of night pains as well. There were no associated comorbidites present like diabetes mellitus and no history of immunosuppresent drugs. She had a history of BCG vaccination after birth.

On examination, tenderness was present over the D12 and L1 regions as well as over the left paraspinal area. There was gibbus formation over D12-L1 spinous processes. The left paraspinal area had fullness from D10 to the sacral level. The neurological examination showed it to be Frankel grade C.

**Figure 1:**
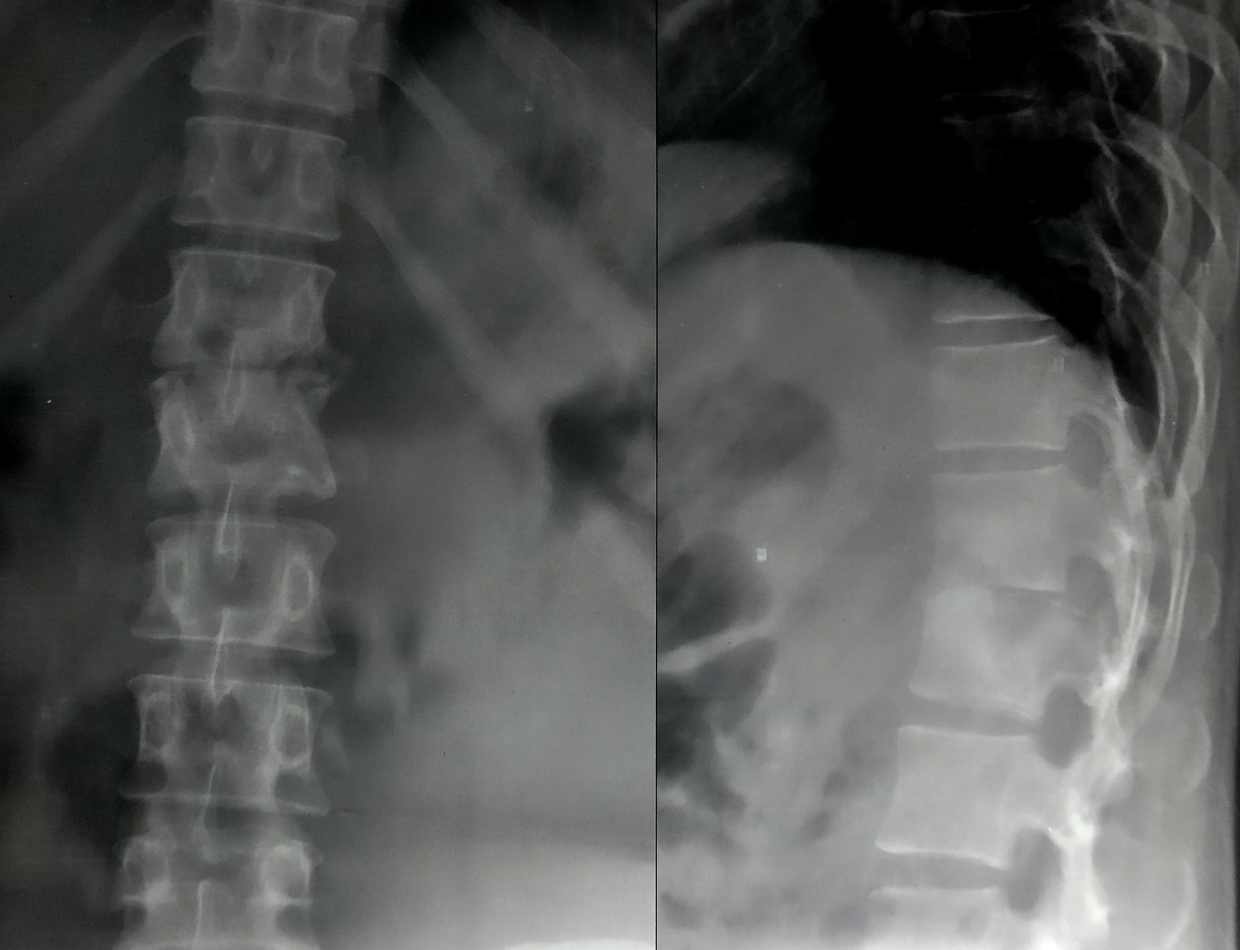
Pre-op radiograph of dorso-lumbar spine (antero-posterior and lateral views) Shows lesion at D12 and L1 vertebrae with kyphotic deformity and destruction of disc space.

Blood investigations were done, which showed an increase in the total WBC count with raised lymphocytes and ESR. Plain radiographs of the dorso-lumbar-sacral spine were obtained, which showed a lesion involving the D12 and L1 vertebral bodies (Figure 1). There was decrease in the intervertebral disc space and wedging of L1 vertebrae with a kyphotic deformity, which suggested tuberculosis.

**Figure 2:**
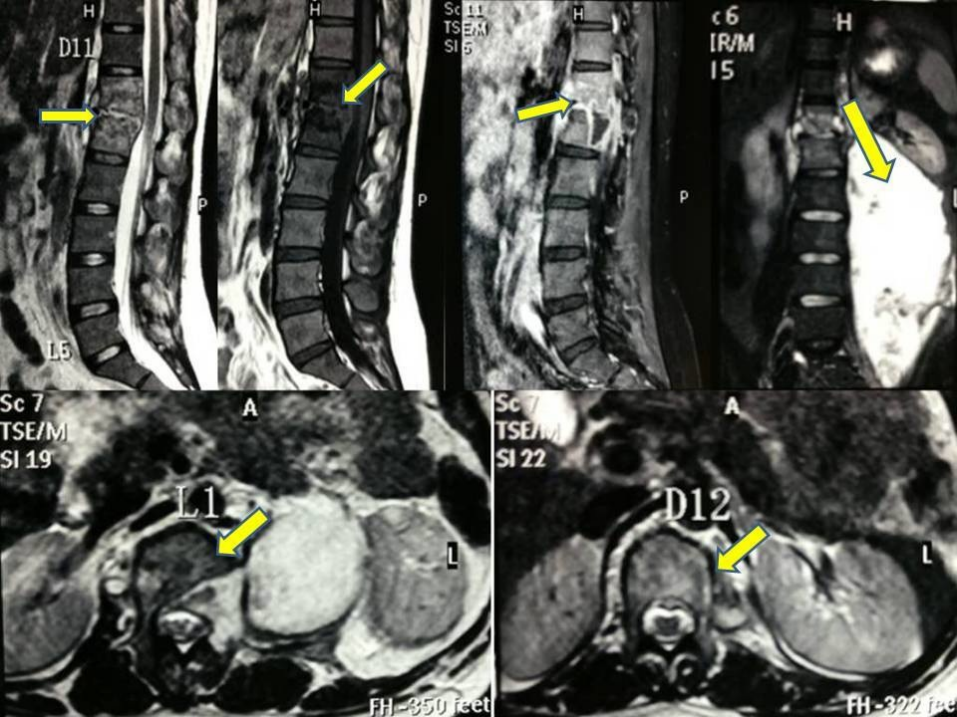
MRI spine (sagittal, coronal and axial cuts) Shows altered marrow signal intensity, hyperintense on T2 and hypointense on T1. There is also a large collection of fluid on the left side iliopsoas region measuring 20.3 cm x 9.2 cm.

An ultrasonography (USG) of the abdomen and pelvis showed a well-defined collection of free floating internal debris in the left lumbar paraspinal region measuring 14 cm x 4.5 cm. A magnetic resonance imaging (MRI) of the dorso-lumbar spine showed a lesion over D12-L1 vertebral bodies with erosion of the disc along with cord and exiting nerve root compression, more on the left side. There was a large collection of fluid on the left iliopsoas region measuring 20.3 cm x 9.2 cm (Figure 2). All these features confirmed the diagnosis of tuberculosis.

Antitubercular drugs (AKT) (isoniazid, rifampicin, ethambutol, pyrazinamide and alternate day streptomycin (IM injection) were given for two weeks, and then the patient was submitted for surgery after obtaining a written informed consent.

**Figure 3:**
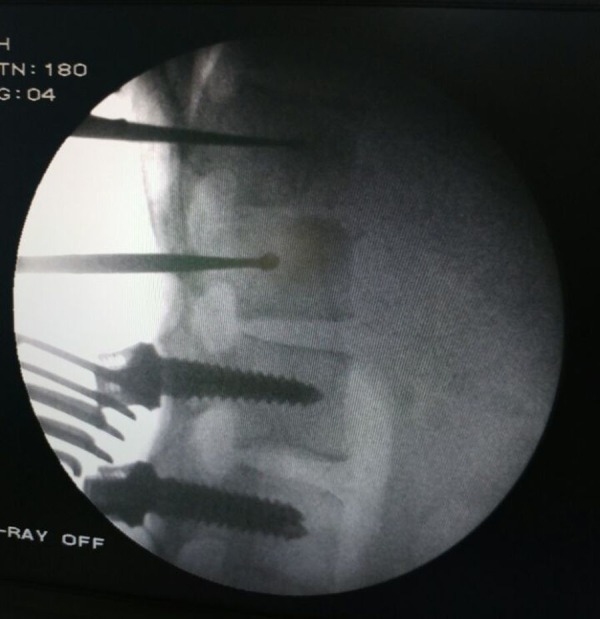
Intra-op IITV image Shows fixation with pedicle screws and transpedicular curettage of the body of L1 vertebra with a curette to remove the tubercular material. IITV - image intensifier television.

A new technique was adopted in which the posterior approach was taken and the D12 and L1 vertebral pedicles were identified and entry was made through the pedicles into the diseased body. The tracts were dilated with sequential screw taps of size 3.5 mm, 4.5 mm, 5.5 mm, and 6.5mm. Curettes were than inserted in the tract created through the pedicles of D12 and L1 into the respective vertebral bodies to remove the diseased tissue and necrotic debris and thus help in debridement and decompression (Figure 3).

**Figure 4:**
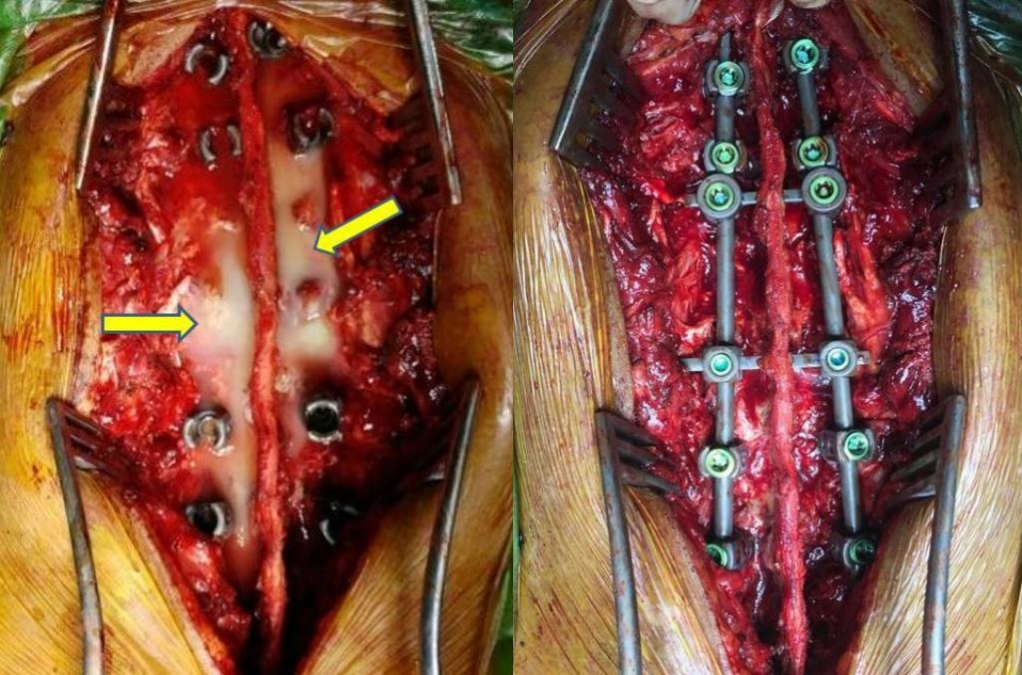
Intra-op clinical picture Shows pus coming out through the tract created after transpedicular curettage and decompression, which was drained out totally (left). Posterior fixation with titanium screws done at two levels above and two levels below the affected vertebrae (right).

Pus started oozing out through the tracts in the pedicles, which suggested that it was multidrug resistant (MDR) TB. As the collection was more on left side, we did transversectomy of L1 vertebra on the left side and about 900 ml of pus was drained out from the iliopsoas abscess (Figure 4). The pus was sent for culture and sensitivity and the caseous material was sent for biopsy, which confirmed it to be tuberculous material.

**Figure 5:**
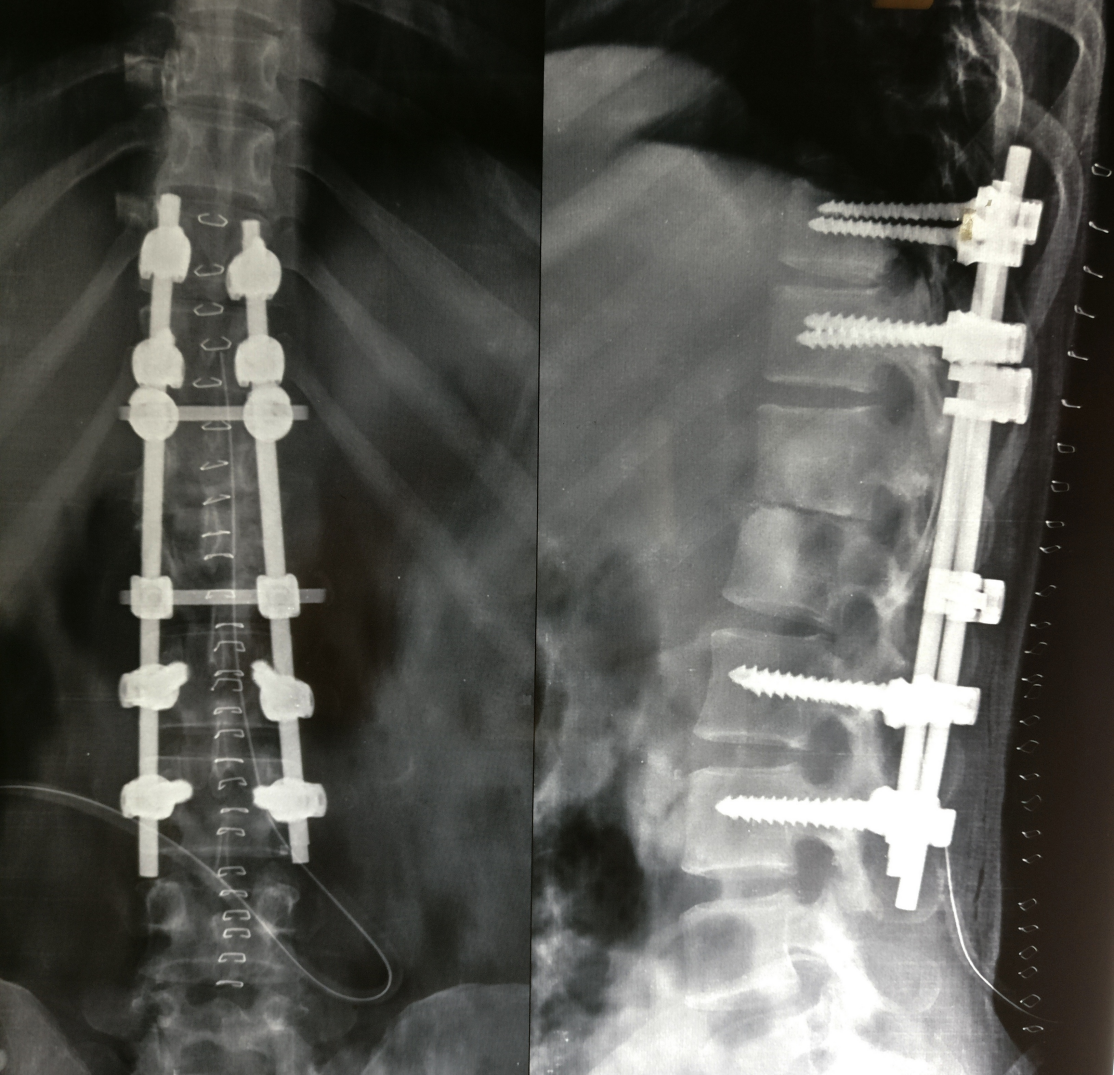
Post-op radiograph of dorso-lumbar spine (antero-posterior and lateral views) Posterior fixation with pedicle screws and rods with two interlinks showing correction of kyphotic deformity.

After this, posterior fixation was done using titanium pedicle screws in D10-11 and L2-3 vertebrae, interconnected with two rods and two interlinks (Figure 5). This helped in correction of the kyphotic deformity and stabilization of the spine.

**Figure 6:**
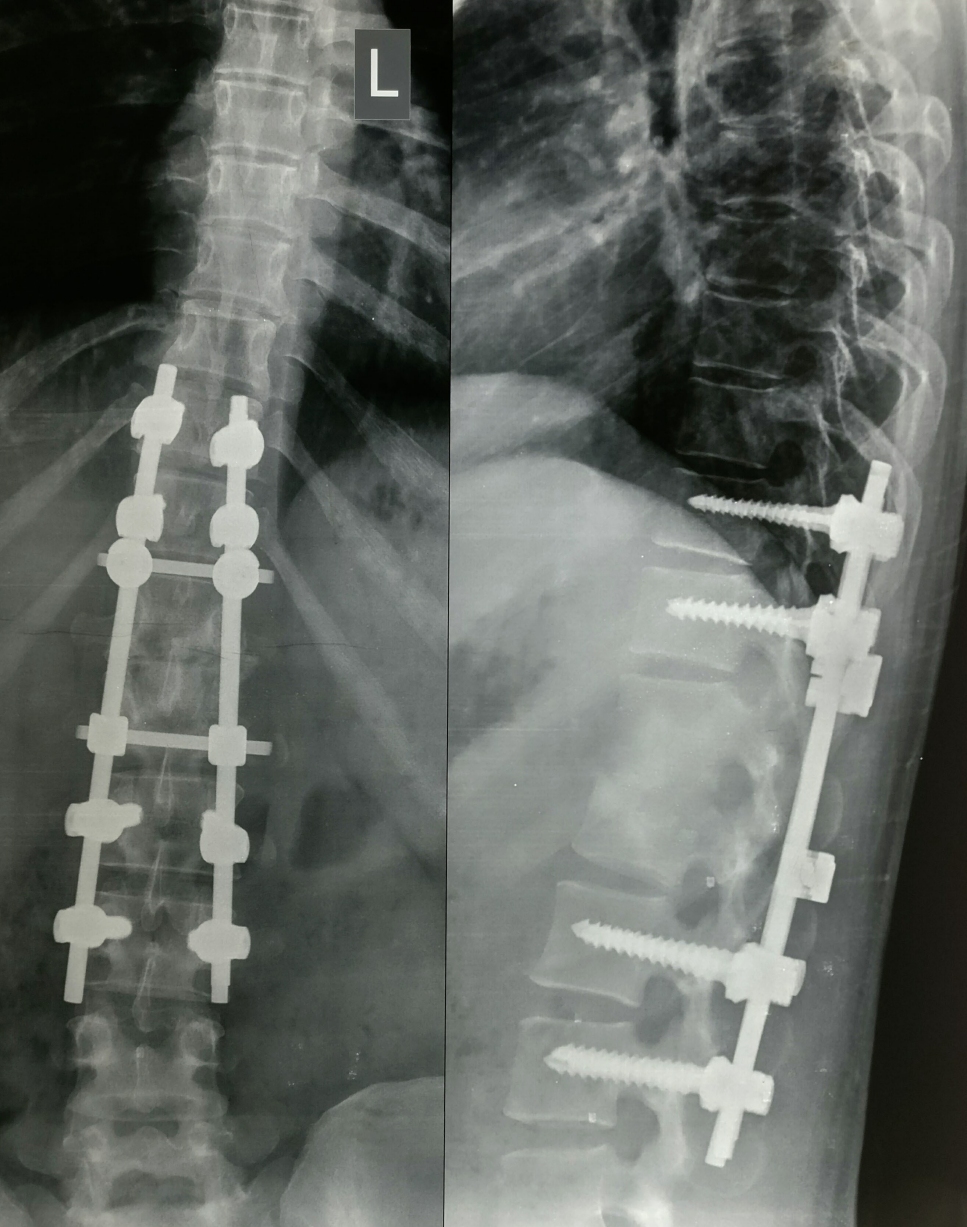
Follow-up radiograph at 22 months Shows fusion of the affected vertebrae and healing of the lesion with sclerotic bone with maintenance of the correction.

Postoperatively, she was kept on bedrest for 15 days and then ambulation was started with ASH (anterior spinal hyperextension) brace for three months. The neurology improved to Frankel grade E by two months. The antitubercular drugs were continued for one year. At final follow-up at 22 months, clinical and radiological features were noted, which showed complete healing and fusion of D12-L1 vertebrae with maintenance of correction on radiographs with no pain and no neurological deficit (Figure 6).

## Discussion

Tuberculosis of the spine is a challenging disease to treat because of the prolonged time of conservative treatment and the technical difficulties of surgical intervention. Patients treated conservatively show an increase in deformity of about 15 degrees. Pan-vertebral lesions, therapeutically refractory disease, severe kyphosis, a developing neurological deficit, lack of improvement or deterioration are indications for surgery, which was also seen in our case [7].

An ideal approach should be one that can provide easy access for complete removal of diseased tissue, not aggravate instability, aid in correction of deformity along with stabilization and fusion, and cause minimal morbidity (blood loss, iatrogenic complications). Traditionally, the anterior approach has been preferred throughout the spine to achieve the above mentioned goals because it offers direct access to the diseased vertebral bodies for debridement and instrumentation. However, the anterior approach has the limitation of being technically very difficult and with a risk of injury to viscera, vascular, and neural structures [8].

Alternatively, a number of posterior approaches have been used to access the anterior vertebral column. These include the transpedicular approach with pedicle subtraction osteotomy, costotransversectomy and antero-lateral decompression [9]. Advantages of the single posterior approach include single staged surgery, avoidance of thoracotomy and a faster recovery period.

We have modified the above described procedures and devised a modified transpedicular approach without pedicle subtraction osteotomy in which through the tract created in the pedicle, the entire vertebral body is curetted out and posterior stabilization is done using instrumentation. We utilize the pedicle as a channel for entry anteriorly into the vertebral body, either unilaterally or bilaterally through the conventional pedicle screw site keeping the lateral pedicle wall intact. Our hypothesis is that once the vertebral body is curetted out, there is hematoma formation in the space created, which gets organised over a period of time into new bone, which leads to fusion as well.

The advantage of this approach is that it preserves the laminae as well as the pedicle, the removal of which can produce instability. This procedure causes less morbidity and significant improvement in pain. It is simple and safe, providing adequate exposure for drainage of paraspinal abscesses as well by doing transversectomy, if required along with the correction of kyphosis. The transpedicular curettage also obliviates the need for fusion as the column is left intact.

## Conclusions

We have used a middle-path approach to treat this condition surgically without the associated comorbidities of the anterior approach. It is a reliable and safe technique to achieve anterior decompression of the spinal canal and posterior stabilization through a single approach and with fewer complications.
